# Comparison of two chromogenic media for the detection of vancomycin-resistant enterococcal carriage by nursing home residents

**DOI:** 10.1016/j.diagmicrobio.2016.04.026

**Published:** 2016-08

**Authors:** Theodore Gouliouris, Beth Blane, Hayley J. Brodrick, Kathy E. Raven, Kirsty E. Ambridge, Angela D. Kidney, Nazreen F. Hadjirin, M. Estée Török, Direk Limmathurotsakul, Sharon J. Peacock

**Affiliations:** a Department of Medicine, University of Cambridge, Cambridge, United Kingdom; b Public Health England, Clinical Microbiology and Public Health Laboratory, Cambridge, United Kingdom; c Cambridge University Hospitals NHS Foundation Trust, Cambridge, United Kingdom; d Department of Veterinary Medicine, University of Cambridge, Cambridge, United Kingdom; e Mahidol-Oxford Tropical Medicine Research Unit, Mahidol University, Bangkok, Thailand; f London School of Hygiene & Tropical Medicine, Keppel Street, London, United Kingdom

**Keywords:** Vancomycin-resistant enterococci, VRE, Enterococcus faecium, Detection, Sensitivity, Selectivity

## Abstract

We compared ChromID VRE and *Brilliance* VRE media for the detection of vancomycin-resistant enterococci (VRE). Using a panel of 28 enterococcal isolates, 10 *vanA Enterococcus faecium* and three *vanA Enterococcus faecalis* isolates grew as per manufacturers’ instructions whilst growth of two *vanC* and eight vancomycin-susceptible enterococci was inhibited on both media. Important differences were noted in the selectivity and chromogenic properties of the two media for *vanA Enterococcus raffinosus* and *vanB E. faecium*. The two media were further evaluated using 295 stool samples from nursing home residents, 34 of which grew VRE (11.5%). ChromID and *Brilliance* had comparable sensitivity, which was increased markedly by prolonging incubation to 48 hours (from 29% to 82%, and from 41% to 85%, respectively) and by a pre-enrichment step (to 97% and 100%, respectively). *Brilliance* VRE agar had higher selectivity at 48 hours, and after pre-enrichment.

## 1 Introduction

Vancomycin-resistant enterococci (VRE) are an important cause of healthcare-associated infections in Europe and USA, mainly attributed to the global dissemination of hospital-adapted lineages of *vanA* mediated vancomycin-resistant *Enterococcus faecium* (VREfm) (Hidron et al., 2008, Lebreton et al., 2013, Werner et al., 2008). In the UK, mandatory surveillance reported 6,246 episodes of VRE bacteremia from 2003 to 2012, with an annual incidence that has failed to decline despite the implementation of infection control interventions that successfully curtailed rates of methicillin-resistant *Staphylococcus aureus* bacteremias and *Clostridium difficile* infections (Gouliouris and Peacock, 2014). VRE are a leading cause of bacteremia in some immunocompromised populations, and are associated with increased mortality and costs compared with infections caused by vancomycin-susceptible enterococci (Butler et al., 2010, DiazGranados et al., 2005, Prematunge et al., 2016).

Screening for fecal carriage of VRE has been advocated as part of a bundle of interventions used to control outbreaks in healthcare settings (Cookson et al., 2006, Siegel et al., 2006), and is routinely used in some units for active surveillance (Brown et al., 2006). Chromogenic media facilitate the early detection of VRE compared to traditional selective media (Asir et al., 2009, Cuzon et al., 2008, Delmas et al., 2007, Grabsch et al., 2008, Jenkins et al., 2011, Peterson et al., 2010), but there is little data comparing the performance of different commercial chromogenic media (Peltroche-Llacsahuanga et al., 2009, Suwantarat et al., 2014). In a recent comparison of five chromogenic media for the detection of VRE in stool, chromID VRE agar (bioMérieux) had the highest sensitivity, although the differences were not statistically significant (Suwantarat et al., 2014). However, *Brilliance* VRE (Oxoid) was not included in this evaluation and chromogenic media were not assessed under different culture conditions such as using pre-enrichment. In addition, no comparative studies have been conducted outside the hospital setting where optimising culture conditions to maximise yield would be important for the accurate determination of VRE stool carriage and epidemiology.

The aims of this method comparison study were to assess the sensitivity and selectivity of chromID VRE and *Brilliance* VRE i) evaluated against a diverse collection of enterococci with previously characterized resistance mechanisms; ii) and tested with or without pre-enrichment using fecal samples from residents of a nursing home in Cambridgeshire.

## 2 Materials and methods

### 2.1 Bacterial isolates

A collection of previously characterized isolates of *E. faecium* (n = 13), *Enterococcus faecalis* (n = 11), *Enterococcus gallinarum*, *Enterococcus casseliflavus*, *Enterococcus raffinosus* and *Enterococcus hirae* was selected for testing. The isolates originated from NCTC collections (n = 12), a retrospective collection of bacteremia isolates from Cambridge University Hospitals (CUH) NHS Foundation Trust (2006–2012, n = 15), and from a meat sample (n = 1) (Table 1). They included isolates with *vanA* and *vanB* acquired resistance, and in the case of *E. faecalis* and *E. faecium* bacteremia isolates, a range of diverse hospital-adapted STs Raven et al., 2016, Raven et al., 2016). The organisms were grown from glycerol bead vials stored at −80°C onto Columbia blood agar (CBA, Oxoid) and 200 μl of a 0.5 McFarland suspension was streaked using the four-quadrant technique onto chromID VRE agar (bioMérieux) and *Brilliance* VRE agar (Oxoid). Growth was assessed after 24 and 48 hours aerobic incubation at 37 °C. Vancomycin minimum inhibitory concentration was measured using Etest (bioMérieux).

### 2.2 Nursing home study setting and design

The study was conducted as part of a larger prospective longitudinal VRE gut carriage survey of residents in a single nursing home in Cambridgeshire (Brodrick et al., 2016). Written informed consent was obtained from participants or consultees, as appropriate. The study protocol was approved by the National Research Ethics Service East of England Ethics Committee (NRES ref: 13/LO/1278), and the CUH Research and Development Department (ref: A093007).

### 2.3 Sample collection, media and culture conditions

A total of 295 stool samples were collected from 37 participants, with a median number of samples per subject of 8 (range 1–14, interquartile range 5–11). Stool samples were collected at study entry and then every week for 14 weeks, starting in March 2014. Stool samples were processed in the laboratory within 24 hours, except for samples obtained over the weekend, which were refrigerated and processed within 72 hours. We performed direct plating onto chromID VRE agar and *Brilliance* VRE agar, and plating onto both media after a selective pre-enrichment step. For direct plating, a pea-sized amount of stool (approximately 0.2 g) was homogenized in 2 ml of Brain Heart Infusion broth (BHIB, Merck), from which 200 μl was inoculated onto each of the chromogenic agar. The remaining homogenate was made up to 5 ml BHIB with a final concentration of 3 mg/L of vancomycin (Sigma) and incubated with shaking at 150 rpm at 37 °C aerobically overnight. Two hundred μl of this broth was then inoculated onto each chromogenic agar. All chromogenic agar plates were incubated at 37 °C in air and read at 24 and 48 hours.

Colonies with an appearance consistent with the two target species vancomycin-resistant *E. faecalis* (VREfs) and VR *E. faecium* (VREfm) as per the manufacturers’ instructions were followed up by subculture of a single colony of each phenotype on CBA with a 5 μg vancomycin disc (Oxoid). Enterococci from subcultures were confirmed to species level using matrix-assisted laser desorption/ionisation time-of-flight mass spectrometry (MALDI-TOF MS, Biotyper 3.1, Bruker Daltonics). Antimicrobial susceptibility testing including vancomycin and teicoplanin was performed using the AST-P607 card on the Vitek 2 instrument (bioMérieux) calibrated against European Committee on Antimicrobial Susceptibility Testing breakpoints (EUCAST, 2014). Colonies with an atypical phenotype were evaluated by Gram stain, microscopy and MALDI-TOF MS as required.

### 2.4 DNA extraction and molecular confirmation of species and van genes

Bacterial species identification, ST, and the presence of *van* genes were confirmed from whole genome sequence data (Brodrick et al., 2016). DNA was extracted using the QIAxtractor (QIAgen), according to the manufacturer’s instructions. Library preparation was conducted according to the Illumina protocol, and sequencing was performed on an Illumina HiSeq2000 with 100-cycle paired-end runs. Molecular confirmation of the *van* genes was sought by *in silico* PCR using published primers (Depardieu et al., 2004, Dutka-Malen et al., 1995).

### 2.5 Statistical analysis

Sensitivity of detection of VRE was calculated by comparing the number of positive plates for a given method with the cumulative yield of VRE from all four methods (direct plating onto two media, and plating onto both following a pre-enrichment step). Selectivity for each method was defined as the number of plates that suppressed the growth of organisms that were not VREfs or VREfm. Statistical analysis was performed using STATA, version 12.1 (STATA, College Station, Texas, USA). Sensitivity and selectivity were compared using the exact McNemar test.

## 3 Results

### 3.1 Evaluation study against characterized enterococcal isolates

The selectivity and chromogenic features of chromID and *Brilliance* were evaluated against a panel of 28 enterococcal isolates (Table 1). Both media inhibited the growth of *vanC* enterococci and vancomycin-susceptible *E. faecium* and *E. faecalis*. Growth of *vanA* VREfs and VREfm was observed for all tested isolates as per manufacturers’ instructions. *vanB* VREfm grew well on chromID but showed poor growth on *Brilliance* (small scanty colonies), particularly at 24 hours. Both media supported the growth of non-target *vanA* VRE species, namely *E. raffinosus* and *E. hirae*. *E. raffinosus* grew as colorless colonies on chromID but as purple colonies resembling VREfm on *Brilliance*. By contrast, *E. hirae* was indistinguishable in appearance from VREfm on both media.

### 3.2 Evaluation of sensitivity and selectivity in nursing home study

Target organisms were isolated from 34/295 (11.5%) stool samples, all of which were ampicillin-resistant *E. faecium* and of the VanA phenotype based on Vitek 2 results and their colonial morphology matched the manufacturers’ description. Presence of the *vanA* gene was confirmed for all 34 isolates. The isolates originated from 3 of 37 residents (8.1%) who were positive on at least one occasion (range 6–14 samples). Four ST types were identified (18, 80, 203, and 787, all belonging to the clonal expansion of clade A).

Detection of VRE was comparable between the two media for all four methods (Table 2, all *P* > 0.10). Sensitivity of detection following direct plating was increased when incubated for the longer duration of 48 hours (from 29% to 82% for chromID agar, *P* < 0.0001 and from 41% to 85% for *Brilliance* agar, *P* = 0.0001). Pre-enrichment with 48 hours incubation further increased the sensitivity of detection of VRE (from 82% to 97% for chromID agar, *P* = 0.06 and from 85% to 100% for *Brilliance* agar, *P* = 0.03). There was a trend for *Brilliance* agar to be more selective than chromID media at 48 hours after direct plating (97% vs. 94%, *P* = 0.05). Selectivity of *Brilliance* agar was significantly higher than that of chromID agar at both 24 hours (95% vs. 88%, *P* = 0.002) and 48 hours (92% vs. 77%, *P* < 0.0001) after pre-enrichment. Selectivity of both plates was poorer at 48 hours versus 24 hours after pre-enrichment (*P* = 0.0002 for *Brilliance* agar, and *P* < 0.0001 for chromID agar).

Gram-negative bacilli were the majority of non-target organisms (false positives) encountered and accounted for most of the difference in selectivity between the two media. Both manufacturers specify that plates can be incubated for up to 48 hours and that a pre-enrichment step can be used. Despite the differences in selectivity, non-target organisms were easily distinguishable from VRE on both media based on color and colonial morphology. However, vancomycin-resistant *Enterococcus avium* was isolated as a colorless colony on chromID from two samples originating from the same patient (but not from *Brilliance*).

Positive and negative predictive values for each culture method before and after taking into account Gram stain results are shown in Supplementary Tables 1 and 2. Specificity and positive predictive value improved significantly for both media after taking into account the Gram stain result.

## 4 Discussion

We compared the performance of chromID and *Brilliance* for the detection of VRE. Testing fecal carriage in a nursing home population in Cambridgeshire, the two media had comparable sensitivity for the isolation of *vanA* VREfm, but *Brilliance* agar had higher selectivity than chromID agar by suppressing growth of Gram-negative organisms. Sensitivity was markedly increased for both media by prolonging the time of incubation to 48 hours after direct plating, and by a pre-enrichment step. A limitation of this study was the absence of VREfs and *vanB* VRE detection in this population, consistent with *vanA* VREfm dominating the UK epidemic (Cookson et al., 2006, Raven et al., 2016, Raven et al., 2016, [under review]), and the small number of positive patients, which reduced the diversity of positive isolates.

The sensitivity of direct culture at 24 hours was low for both media. Previous studies report sensitivities of over 90% at 24 hours using VRE chromogenic media (Grabsch et al., 2008, Jenkins et al., 2011), but did not use pre-enrichment as a comparator, or studied hospitalized populations such as those in intensive care units where high load of VRE carriage may be expected. By contrast, two studies that used an additional pre-enrichment step to evaluate chromID for the detection of VRE in hospitalized patients also found a low sensitivity at 24 hours of around 50% (Asir et al., 2009, Delmas et al., 2007). Decisions over time of incubation and the use of pre-enrichment for a given application will be influenced by the relative importance of turn-around time versus sensitivity and selectivity of detection, and will depend on population factors such as fecal carriage load.

We found significant differences in the performance of chromID and *Brilliance* using rare *vanA* VRE species (non-faecalis or -faecium) and *vanB* VREfm. Both media supported the growth of *vanA E. raffinosus* and *E. hirae*, however the former could have been missed on chromID as it appeared colorless. Conversely, *vanA E. avium* only grew on chromID. Whilst the occurrence of these organisms is rare, they can potentially have a pathogenic role or act as sources of vancomycin resistance genes. Wijesuriya et al. (2014) compared 4 chromogenic media including chromID and *Brilliance* in a small Australian study where stool samples were spiked predominantly with *vanB* VREfm. *Brilliance* appeared to have lower sensitivity compared to other media in detecting VRE, particularly for those isolates where the vancomycin MIC value was <16 mg/L. These results contrast with the findings of Klare et al. (2012) who reported similar performance between VRE *Brilliance* and chromID against a panel of 129 *vanB E. faecium* isolates. We found that the two *vanB* VREfm isolates with different vancomycin MICs (24 and >256 mg/L) produced slower growth on VRE *Brilliance* compared to chromID. This effect could not be explained solely by the MIC values, as the *vanB* VREfs isolate which had a lower MIC (16 mg/L) grew well on both media. We would caution against using *Brilliance* in a setting where VREfm is predominantly mediated by *vanB* until further comparative data is available from clinical studies.

In conclusion, the combination of highest sensitivity and selectivity was achieved by *Brilliance* agar with a pre-enrichment step and 48 hours incubation after plating when evaluated in a nursing home population with predominantly *vanA* VREfm carriage.

## Figures and Tables

**Table 1:** Performance of two chromogenic agars tested against a panel of 28 diverse enterococci.

Isolate	Species	*van* gene	Vancomycin MIC	ST type	ChromID VRE		*Brilliance* VRE	
24 hours	48 hours	24 hours	48 hours
EC0041	*E. faecium*	*vanA*	>256	17	+	+	+	+
EC0179	*E. faecium*	*vanA*	>256	64	+	+	+	+
EC0212	*E. faecium*	*vanA*	>256	78	+	+	+	+
EC0180	*E. faecium*	*vanA*	>256	117	+	+	+	+
EC0115	*E. faecium*	*vanA*	>256	202	+	+	+	+
EC0051	*E. faecium*	*vanA*	>256	203	+	+	+	+
EC0518	*E. faecium*	*vanA*	>256	375	+	+	+	+
EC0220	*E. faecium*	*vanA*	>256	412	+	+	+	+
EC0176	*E. faecium*	*vanA*	>256	552	+	+	+	+
NCTC12202	*E. faecium*	*vanA*	>256	18	+	+	+	+
EC0050	*E. faecalis*	*vanA*	>256	103	+	+	+	+
NCTC12201	*E. faecalis*	*vanA*	>256	28	+	+	+	+
NCTC12203	*E. faecalis*	*vanA*	>256	6	+	+	+	+
EC0109	*E. raffinosus*	*vanA*	>256		colorless +	colorless +	+	+
VREN1021	*E. hirae*	*vanA*	128		+	+	+	+
EC0181	*E. faecium*	*vanB*	>256	203	+	+	very poor	poor
NCTC12952	*E. faecium*	*vanB*	24	18	+	+	very poor	poor
NCTC13379	*E. faecalis*	*vanB*	16	6	+	+	+	+
NCTC12362	*E. casseliflavus*	*vanC*	3		-	-	-	-
NCTC12360	*E. gallinarum*	*vanC*	4		-	-	-	-
NCTC7378	*E. faecium*	-	0.75	361	-	-	-	-
EC0439	*E. faecalis*	-	1.5	6	-	-	-	-
EC0441	*E. faecalis*	-	1	103	-	-	-	-
EC1246	*E. faecalis*	-	1	28	-	-	-	-
NCTC8131	*E. faecalis*	-	2	21	-	-	-	-
NCTC775	*E. faecalis*	-	0.38	25	-	-	-	-
NCTC8175	*E. faecalis*	-	2	40	-	-	-	-
NCTC8132	*E. faecalis*	-	2	55	-	-	-	-

Growth was quantified as + (growth in all four quadrants), poor (scanty growth in more than one quadrant) and very poor (scanty growth on primary inoculum only). Colonial appearance was as per manufacturers’ instructions for *E. faecalis* and *E. faecium*. For other species, the colonial morphology was similar to *E. faecium* unless stated otherwise.

MIC, minimum inhibitory concentration (mg/L).

**Table 2:** Sensitivity and selectivity of two chromogenic agars for the isolation of vancomycin-resistant enterococci from 295 stool samples.

	ChromID VRE direct plating Method 1		*Brilliance* VRE direct plating Method 2		ChromID VRE plating after enrichment Method 3		*Brilliance* VRE plating after enrichment Method 4	
Incubation time (hours)	24	48	24	48	24	48	24	48
Number of plates growing VRE^a^	10	28	14	29	32	33	33	34
Sensitivity^b^	29%	82%	41%	85%	94%	97%	97%	100%
Number of plates NOT growing organisms other than VRE^a^	292	277	293	285	259	227	280	270
Selectivity^c^	99%	94%	99%	97%	88%	77%	95%	92%
Number of plates growing Gram-negative organisms	3	10	2	5	28	58	11	18
Number of plates growing Gram-positive organisms	0	6	0	4	6	7	4	6
Number of plates growing yeasts	0	2	0	1	2	3	0	1

VRE = vancomycin-resistant enterococci.

a Target VRE organisms consisted of vancomycin-resistant *E. faecalis* (VREfs) and vancomycin-resistant *E. faecium* (VREfm).

b Sensitivity was calculated by comparing the number of positive plates for a given method, compared with the cumulative yield of VREfs or VREfm from all four methods (direct plating onto two media, and plating onto both following a pre-enrichment step, n = 34).

c Selectivity for each method was defined as the number of plates that suppressed the growth of organisms that were not VREfs or VREfm (out of all 295 samples).

## Appendix A Supplementary data

Supplementary Table 1. Sensitivity, specificity, PPV and NPV for chromID VRE and VRE *Brilliance* after 24 and 48 hours incubation, with and without pre-enrichment. Supplementary Table 2. Sensitivity, specificity, PPV and NPV for chromID VRE and VRE *Brilliance* after 24 and 48 hours incubation, with and without pre-enrichment, following Gram staining.
