# Supplementary material for: Comparison of two chromogenic media for the detection of vancomycin-resistant enterococcal carriage by nursing home residents
Source: Diagn Microbiol Infect Dis. 2016 Aug;85(4):409–12. doi: 10.1016/j.diagmicrobio.2016.04.026 (PMC4950902; doi:10.1016/j.diagmicrobio.2016.04.026)
Supplement: Supplementary file 1 — Supplementary Table 1. Sensitivity, specificity, PPV and NPV for chromID VRE and VRE Brilliance after 24 and 48 hours incubation, with and without pre-enrichment. Supplementary Table 2. Sensitivity, specificity, PPV and NPV for chromID VRE and VRE Brilliance after 24 and 48 hours incubation, with and without pre-enrichment, following Gram staining. [file mmc1.docx]

Supplementary Table 1. Sensitivity, specificity, PPV and NPV for chromID VRE and VRE *Brilliance* after 24 and 48 hours incubation, with and without pre-enrichment.

| Method | Incubation time (hours) | TP | FP | TN | FN | Se (%) | Sp (%) | PPV (%) | NPV (%) |
| --- | --- | --- | --- | --- | --- | --- | --- | --- | --- |
| Direct | | | | | | | | | |
| ChromID VRE | 24 | 10 | 2 | 259 | 24 | 29.4 | 99.2 | 83.3 | 91.5 |
|  | 48 | 28 | 15 | 246 | 6 | 82.4 | 94.3 | 65.1 | 97.6 |
| *Brilliance* VRE | 24 | 14 | 2 | 259 | 20 | 41.2 | 99.2 | 87.5 | 92.8 |
|  | 48 | 29 | 8 | 253 | 5 | 85.3 | 96.9 | 78.4 | 98.1 |
| Enrichment | | | | | | | | | |
| ChromID VRE | 24 | 32 | 33 | 228 | 2 | 94.1 | 87.4 | 49.2 | 99.1 |
|  | 48 | 33 | 61 | 200 | 1 | 97.1 | 76.6 | 35.1 | 99.5 |
| *Brilliance* VRE | 24 | 33 | 14 | 247 | 1 | 97.1 | 94.6 | 70.2 | 99.6 |
|  | 48 | 34 | 24 | 237 | 0 | 100 | 90.8 | 58.6 | 100 |

Supplementary Table 2. Sensitivity, specificity, PPV and NPV for chromID VRE and VRE *Brilliance* after 24 and 48 hours incubation, with and without pre-enrichment, following Gram staining.

| Method | Incubation time (hours) | TP | FP | TN | FN | Se (%) | Sp (%) | PPV (%) | NPV (%) |
| --- | --- | --- | --- | --- | --- | --- | --- | --- | --- |
| Direct | | | | | | | | | |
| ChromID VRE | 24 | 10 | 0 | 261 | 24 | 29.4 | 100 | 100 | 91.6 |
|  | 48 | 28 | 5 | 256 | 6 | 82.4 | 98.1 | 84.8 | 97.7 |
| *Brilliance* VRE | 24 | 14 | 0 | 261 | 20 | 41.2 | 100 | 100 | 92.9 |
|  | 48 | 29 | 2 | 259 | 5 | 85.3 | 99.2 | 93.5 | 98.1 |
| Enrichment | | | | | | | | | |
| ChromID VRE | 24 | 32 | 5 | 256 | 2 | 94.1 | 98.1 | 86.5 | 99.2 |
|  | 48 | 33 | 6 | 255 | 1 | 97.1 | 97.7 | 84.6 | 99.6 |
| *Brilliance* VRE | 24 | 33 | 3 | 258 | 1 | 97.1 | 98.9 | 91.7 | 99.6 |
|  | 48 | 34 | 5 | 256 | 0 | 100 | 98.1 | 87.2 | 100 |

TP, true positive; FP, false positive; TN, true negative; FN, false negative; Se, sensitivity; Sp, specificity; PPV, positive predictive value; NPV, negative predictive value.
